# The *Schizosaccharomyces pombe* ornithine-N^5^-oxygenase Sib2 interacts with the N^5^-transacetylase Sib3 in the ferrichrome biosynthetic pathway

**DOI:** 10.3389/fmicb.2024.1467397

**Published:** 2024-09-10

**Authors:** Berthy Mbuya, Samuel Plante, Farouk Ammar, Ariane Brault, Simon Labbé

**Affiliations:** Département de Biochimie et de Génomique Fonctionnelle, Faculté de Médecine et des Sciences de la Santé, Université de Sherbrooke, Sherbrooke, QC, Canada

**Keywords:** siderophore, ferrichrome, cross-feeding, budding yeast, fission yeast

## Abstract

The fission yeast *Schizosaccharomyces pombe* produces the hydroxamate-type siderophore ferrichrome (Fc). The biosynthesis of Fc requires the Fc synthase Sib1, the ornithine-N^5^-oxygenase Sib2, and the N^5^-hydroxyornithine-N^5^-transacetylase Sib3. In this study, we demonstrate the critical importance of the His^248^ residue of Sib3 in Fc production. Cells expressing a *sib3H248A* mutant allele fail to grow in iron-poor media without Fc supplementation. These *sib3H248A* mutant cells are consistently unable to promote Fc-dependent growth of *Saccharomyces cerevisiae* cells in cross-feeding experiments. Green fluorescent protein (GFP)-tagged wild-type Sib3 and mutant Sib3H248A exhibit a pancellular distribution. Coimmunoprecipitation assays revealed that both wild-type and Sib3H248A physically interact with Sib2. Further analysis identified a minimal C-terminal region from amino acids 290–334 of Sib3 that is required for interaction with Sib2. Deletion mapping analysis identified two regions of Sib2 as being required for its association with Sib3. The first region encompasses amino acids 1–135, and the second region corresponds to amino acids 281–358 of Sib2. Taken together, these results describe the first example of a physical interaction between an ornithine-N^5^-oxygenase and an N^5^-hydroxyornithine-N^5^-transacetylase controlling the biosynthesis of a hydroxamate-type siderophore.

## Introduction

Iron is a vital micronutrient for all eukaryotes. Its distinctive ability to exist in two redox states makes it a crucial cofactor at the active centers of numerous enzymes (Katsarou and Pantopoulos, 2020). These iron-dependent enzymes play key roles in fundamental cellular processes, such as oxidative phosphorylation, amino acid biosynthesis, and lipid metabolism (Puig et al., 2017; Galaris et al., 2019; Philpott et al., 2020). One notable characteristic of iron is its poor bioavailability at physiological pH under atmospheric oxygen conditions. This is due to its redox activity, which promotes its conversion into insoluble ferric hydroxide forms in the environment (Aguiar et al., 2021). Under this context of limited iron availability, organisms have evolved various mechanisms to acquire iron from diverse sources. One such mechanism involves the synthesis and secretion of low-molecular-mass siderophores that specifically bind ferric iron with an extremely high affinity (Haas et al., 2008; Hider and Kong, 2010; Haas, 2014). Once they associate with extracellular ferric iron, siderophore-iron complexes (holo-siderophores) are captured by the producer microbe or by other neighboring opportunistic microorganism species (Seth and Taga, 2014; Pande and Kost, 2017; D'Souza et al., 2018; Fritts et al., 2021). When non-producing microbes take up holo-siderophores, a cross-feeding interaction between the producer and neighboring microbes can be established without incurring any deleterious fitness costs. In this one-way cross-feeding context, a donor microbe secretes a siderophore without a fitness cost, which is then utilized by a recipient microbe unable of synthesizing or secreting this siderophore (Joshi et al., 2006; Galet et al., 2015; Butaitė et al., 2017; Fritts et al., 2021). The producer of the same siderophore can also use its siderophore uptake machinery to capture holo-siderophores produced by neighboring microorganisms, thereby conserving energy that would otherwise be spent on synthesizing siderophores.

The fission yeast *Schizosaccharomyces pombe* synthesizes and secretes the hydroxamate-type siderophore ferrichrome (Fc) under conditions of iron starvation (Schrettl et al., 2004a; Mercier and Labbé, 2010; Plante and Labbé, 2019). Upon secretion, Fc can bind to ferric iron in the environment. Fc-iron complexes (holo-Fc) are subsequently captured by Str1, a cell-surface siderophore transporter. Str1 belongs to a subfamily of the major facilitator superfamily of transporters and specifically transports Fc-bound iron across the plasma membrane of *S. pombe* (Plante and Labbé, 2019). Alternatively, holo-Fc are taken up by specific cell-surface transporters expressed in opportunistic microbes that are unable to synthesize Fc themselves. Among potential neighboring microbial interactors, *Saccharomyces cerevisiae* can utilize Fc produced by *S. pombe* to support its growth when Fc-iron serves as the sole iron source in a one-way cross-feeding context (Brault et al., 2022).

The synthesis of Fc in *S. pombe* relies on three proteins. The first enzyme is the ornithine-N^5^-oxygenase Sib2, which catalyzes the N^5^ hydroxylation of ornithine. The second step is mediated by the N^5^-hydroxyornithine-N^5^-transacetylase Sib3, which is predicted to catalyze the acetylation of N^5^-hydroxyornithine. Lastly, the resulting N^5^-acetyl-N^5^-hydroxyornithine product, along with three glycine residues, is used to assemble the final Fc product via the activity of the non-ribosomal peptide synthetase (NRPS) Sib1 (Schrettl et al., 2004b; Brault et al., 2022). Microscopic studies using functional fluorescently tagged Sib1, Sib2, and Sib3 proteins have shown that all three proteins can be found in the cytoplasm when cells are cultured under low-iron conditions (Brault et al., 2022). Furthermore, analysis of protein–protein interaction by coimmunoprecipitation assays revealed that Sib2 and Sib3 interact with one another under conditions of iron starvation (Brault et al., 2022).

Transcription of the genes *sib1^+^*, *sib2^+^*, and *str1^+^* is regulated in response to changes in iron levels. These three genes are transcriptionally induced under low environmental iron conditions, whereas their expression is repressed under iron-replete conditions (Pelletier et al., 2003; Plante and Labbé, 2019; Brault et al., 2022). The iron-mediated transcriptional repression of *sib1^+^*, *sib2^+^*, and *str1^+^* mRNA levels is regulated by the iron-regulatory GATA-type repressor Fep1 (Pelletier et al., 2002; Pelletier et al., 2003; Mercier and Labbé, 2010; Plante and Labbé, 2019; Brault et al., 2022). Conversely, in cells transitioning from high to low iron levels, Fep1 loses its ability to bind to its GATA regulatory elements on chromatin, resulting in the activation of target gene expression (Jbel et al., 2009). In the case of *sib3^+^*, its expression is constitutive and not regulated by iron or Fep1 (Brault et al., 2022).

Our previous studies established that *sib3Δ* mutant cells are unable to produce Fc and exhibit severe growth defects in iron-poor media (Brault et al., 2022). Based on comparative analysis inferred from sequence similarity, Sib3 belongs to the GCN5-related N-acetyltransferase (GNAT) family of acyltransferases (Card et al., 2005; Rutherford et al., 2024). The GNAT superfamily comprises numerous enzymes, many of which catalyze the transfer of an acetyl group from acetyl-CoA to the primary amine of acceptor substrates. Interestingly, a sub-group of acetyltransferase enzymes involved in siderophore biosynthesis share sequence homology with GNAT proteins (Blatzer et al., 2011). In *Escherichia coli*, the acetyltransferase IucB catalyzes the N-acetylation of N^6^-hydroxylysine, which is involved in the second step of aerobactin siderophore biosynthesis (de Lorenzo et al., 1986). In *Bordetella bronchiseptica*, the N-acetyltransferase AlcB is predicted to acetylate the hydroxylamine group of N-hydroxyputrescine during the biogenesis of the alcaligen siderophore (Challis, 2005). Mechanistic analysis of *Mycobacterium tuberculosis* Rv1347c protein revealed that this lysine N^6^-acetyltransferase is required for the N-acetylation of a precursor involved in mycobactin siderophore production (Krithika et al., 2006; Frankel and Blanchard, 2008). In the case of the fungal siderophore-biosynthetic acetyltransferase SidL from *Aspergillus fumigatus*, it is required for the acetylation of N^5^-hydroxyornithine to N^5^-acetyl-N^5^-hydroxyornithine in the second step of ferricrocin siderophore biosynthesis (Blatzer et al., 2011). In addition to SidL, *A. fumigatus* possesses a second N^5^-transacylase, SidF (Schrettl et al., 2007; Grundlinger et al., 2013). Although this enzyme localizes in peroxisomes and is required for the biosynthesis of fusarinine-type siderophores, its C-terminal region contains a GNAT-like domain, which exhibits similarity to SidL and other GNAT-like proteins involved in siderophore biosynthesis. In the fungus *Ustilago maydis*, Fer5 functions as a hydroxyornithine acetyltransferase for the generation of methylglutaconyl hydroxyornithine, an essential precursor for ferrichrome A biosynthesis (Winterberg et al., 2010). A common feature of these aforementioned proteins is the presence of a conserved domain, denoted MbtK/IucB, which predicts a functional acetyltransferase activity involves in siderophore biosynthesis. In the case of *S. pombe* Sib3, proteomic analysis predicted the presence of an MbtK/IucB-like domain encompassing amino acids 162–209 of Sib3 (Rutherford et al., 2024).

In the present study, we demonstrate that the residue His^248^ of Sib3 is essential for the growth of *S. pombe* cells under low-iron conditions when Fc-bound iron is the sole iron source. Consequently, *ARN1*-expressing *S. cerevisiae fet3Δ arn1-4Δ* cells are unable to grow in the vicinity of *S. pombe sib3H248A* mutant cells, which are defective in Fc biosynthesis to fuel the growth of the recipient *S. cerevisiae fet3Δ arn1-4Δ ARN1* cells. Coimmunoprecipitation assays show that Sib3H248A and Sib3E286A mutants still interact with Sib2. Further protein–protein interaction analysis reveals that two regions, spanning amino acids 1–135 and 281–358 of Sib2, are required for interaction with Sib3. Deletion mapping analysis indicates that the Sib3 C terminus, comprising amino acids 290–334, is sufficient for Sib2-Sib3 association. Collectively, the functional dissection of Sib3 identifies residue H248 as critical for Fc production and a short C-terminal region of Sib3 as necessary for its interaction with Sib2.

## Materials and methods

### Strains and culture conditions

The genotypic characteristics of the *S. pombe* and *S. cerevisiae* strains used in this study are detailed in Table 1. *Schizosaccharomyces pombe* strains were cultured on yeast extract with supplements (YES) medium under non-selective growth conditions, following established protocols (Sabatinos and Forsburg, 2010). For *S. pombe* strains requiring plasmid integration or transformation, synthetic Edinburgh minimal medium (EMM) lacking a specific ribonucleotide base or specific amino acids was used to allow the selection a particular plasmid in transformed yeast cells. For the selection of *S. pombe* transformants via geneticin (G418) resistance, 200 mg/L of G418 was added to YES medium. Yeast extract (1%), bactopeptone (2%), and dextrose (2%) (YPD) medium was used for the routine growth of *S. cerevisiae* strains. Synthetic dropout (SC) medium was utilized when DNA plasmid transformation was required in *S. cerevisiae* cells (Yun et al., 2000; Sherman, 2002). A modified synthetic dextrose minimal medium depleted for copper and iron (SD^-Cu-Fe^) was used for cross-feeding experiments (Brault et al., 2022). To perform cross-feeding assays, aliquots of the indicated cultures of *S. pombe* (1 × 10^7^ cells/10 μL) and *S. cerevisiae* (3 × 10^3^ cells/10 μL) strains were spotted in the vicinity of one another as previously described (Brault et al., 2022).

**Table 1:** Yeast strains used in this study.

*S. pombe* strain	Genotype	Source
FY435	*h^+^ his7-366 leu1-32 ura4-*∆*18 ade6-M210*	Pelletier et al. (2002)
*fep1*Δ	*h^+^ his7-366 leu1-32 ura4-*∆*18 ade6-M210 fep1*∆*::ura4^+^*	Pelletier et al. (2002)
AMY58	*h^+^ his7-366 leu1-32 ura4-*∆*18 ade6-M210 sib1*∆ *sib2Δ::KAN^r^*	Mercier and Labbé (2010)
ABY151	*h^+^ his7-366 leu1-32 ura4-*∆*18 ade6-M210 sib2*∆*::KAN^r^*	Brault et al. (2022)
ABY127	*h^+^ his7-366 leu1-32 ura4-*∆*18 ade6-M210 sib3*∆*::KAN^r^*	Brault et al. (2022)
BMY1	*h^+^ his7-366 leu1-32 ura4-*∆*18 ade6-M210 sib3*∆*::loxP sib2∆::KAN^r^*	This study
***S. cerevisiae* strain**	**Genotype**	**Source**
YPH499	*MAT*a *ura3-52 lys2-801 ade2-101 trp1-63*Δ *his3-200∆ leu2-1∆*	Yun et al. (2000)
*fet3*Δ *arn1-4*Δ	*MAT*a *ura3-52 lys2-801 ade2-101 trp1-63Δ his3-200∆ leu2-1∆ fet3*Δ*::HIS3 arn1Δ::HISG arn2Δ::HISG arn3Δ::HISG arn4Δ::HISG-URA3-HISG*	Yun et al. (2000)

### DNA constructs

Plasmids pJK*sib3^+^-GFP*, pJK*sib3^+^-TAP*, and pSP1*sib3^+^-TAP* have been previously described (Brault et al., 2022). Plasmid pJK*sib3^+^-GFP* served as the template for introducing site-specific mutations using a PCR overlap extension method (Ho et al., 1989). In the case of pJK*sib3H248A-GFP*, the *sib3^+^* codon corresponding to His^248^ was substituted with an alanine codon. Similarly, the codon corresponding to Glu^286^ was mutated either using pJK*sib3^+^-GFP* or pJK*sib3H248A-GFP* as the template. The codon was replaced with a nucleotide triplet encoding an alanine residue, and the resulting plasmids were denoted as pJK*sib3E286A-GFP* and pJK*sib3H248A/E286A-GFP*, respectively. An identical PCR site-directed mutagenesis procedure was used to generate a series of mutants, this time employing either pJK*sib3^+^-TAP* or pSP1*sib3^+^-TAP* as templates. The resulting plasmids were designated as pJK*sib3H248A-TAP*, pJK*sib3E286A-TAP*, pJK*sib3H248A/E286A-TAP*, pSP1*sib3H248A-TAP*, pSP1*sib3E286A-TAP*, and pSP1*sib3H248A/E286A-TAP*.

To generate a deletion from the C-terminal end of Sib3, the *sib3^+^* gene was isolated by PCR using primers amplifying the *sib3^+^* locus starting at −501 bp from the start codon up to the codon 210 of the gene. This 1,143-bp ApaI-XmaI PCR-amplified DNA segment was inserted into the corresponding sites of pSP1 (Cottarel et al., 1993), creating plasmid pSP1-501sib3codons1–210nostop. The TAP sequence was isolated by PCR, digested with XmaI and SacI, and then insert in-frame into the *sib3* coding region (residues 1–210). This new plasmid was denoted as pSP1-501sib3codons1-210-TAP.

The *sib3^+^* promoter, containing 501-bp, was inserted into the ApaI and EcoRI sites of pSP1, resulting in plasmid pSP1-501*sib3prom*. To create truncated versions of Sib3 from the N-terminal end, four DNA fragments containing C-terminal codons 135–334, 165–334, 211–334, and 290–334 were PCR-amplified with an initiation codon specifying methionine and cloned into the EcoRI and XmaI sites of pSP1-501*sib3prom*. Following the aforementioned procedure, the XmaI-SacI TAP-encoded DNA fragment was inserted in-frame with the 3′-terminal coding sequence of truncated versions of Sib3. The resulting plasmids were denoted as pSP1-501sib3codons135-334-TAP, pSP1-501sib3codons165-334-TAP, pSP1-501sib3codons211-334-TAP, and pSP1-501sib3codons290-334-TAP, respectively.

The plasmid pJB1tp*sib2^+^-GFP* has been previously described (Brault et al., 2022). Three DNA fragments containing distinct N-terminal regions of Sib2 were generated by PCR amplification. After purification, these PCR products were digested with EcoRV and XmaI, and then used to replace the corresponding wild-type *sib2^+^* DNA segment in pJB1tp*sib2^+^-GFP*. The resulting plasmids were designated as pJB1tpsib2codons1-349-GFP, pJB1tpsib2codons1-279-GFP, and pJB1tpsib2codons1-135-GFP, respectively. Three additional PCR-amplified fragments were generated, each containing different C-terminal regions of Sib2. An identical molecular cloning strategy, as described for the C-terminal truncated versions of Sib2, was employed, with the exception that the EcoRV-XmaI fragments of the *sib2^+^* gene possessed an initiator codon methionine in-frame with different 5′-terminal coding sequences of *sib2^+^*. The resulting plasmids were denoted as pJB1tpsib2codons82-431-GFP, pJB1tpsib2codons174-431-GFP, and pJB1tpsib2codons359-431-GFP, respectively. Two additional plasmid constructs, encompassing a central region of Sib2, were designated as pJB1tpsib2codons149-345-GFP and pJB1tpsib2codons281-358-GFP. These central regions were amplified by PCR using primers designed to generate EcoRV and XmaI restriction sites at the upstream and the downstream termini of a sequence spanning 196 or 77 codons within the middle region of *sib2^+^*. Moreover, the sense primer contained an initiator codon in-frame with the coding sequences of the middle region (codons 149–345 or 281–358) of *sib2^+^*. Subsequently, the EcoRV-XmaI DNA fragments were swapped with the corresponding *sib2^+^* EcoRV-XmaI fragment in the pJB1tpsib2codons82-431-GFP plasmid. Plasmids p415GPDARN1-GFP and pBPnmt41x-GFP, used for cross-feeding assays and coimmunoprecipitation experiments, respectively, have been previously described (Brault and Labbé, 2020; Brault et al., 2022).

### Protein extraction and western blot assays

To assess protein steady-state levels, cell lysates were prepared by glass bead disruption using an extraction buffer containing 50 mM Tris–HCl (pH 7.5), 150 mM NaCl, 5 mM MgCl_2_, 0.1% Nonidet P-40, and a complete protease inhibitor mixture (Sigma-Aldrich, P8340). Cell lysis was achieved using a FastPrep-24 instrument (MP Biomedicals, Solon, OH). Equal amounts of each protein sample were resolved by electrophoresis on 8% sodium dodecyl sulfate (SDS)-polyacrylamide gels. Protein transfer from gel to membrane in Western blot assays was carried out as described previously (Ioannoni et al., 2016). The immunodetection of Sib3-TAP, Sib3-GFP, Sib2-GFP, and α-tubulin was performed using polyclonal anti-mouse IgG antibody (ICN Biomedicals), monoclonal anti-GFP antibody B-2 (Santa Cruz Biotechnology), and monoclonal anti-α-tubulin antibody (clone B-5-1-2, Sigma-Aldrich). Subsequently, the membranes were washed and incubated with the appropriate horseradish peroxidase-conjugated secondary antibodies (Amersham Biosciences), developed using enhanced chemiluminescence (ECL) reagents, and visualized via chemiluminescence using an ImageQuant LAS 4000 instrument (GE Healthcare) equipped with a Fujifilm High Sensitivity F0.85 43 mm camera.

### Pull-down assays

Protein–protein interaction assays were performed to further delineate the regions of Sib2 and Sib3 necessary for their mutual interaction. Full-length or truncated versions of Sib2-GFP were coexpressed with Sib3-TAP in *sib2Δ sib3Δ* cells. Similarly, *sib2Δ sib3Δ* cells were used to coexpress full-length Sib2-GFP with wild-type Sib3-TAP or its mutant derivatives. For each indicated pair of coexpressed proteins, cotransformed *sib2Δ sib3Δ* cells were grown in EMM medium until mid-logarithmic phase. At this point, cells were incubated with Dip (250 μM) and L-ornithine (15 mM) (Sigma-Aldrich) for 2 h. Subsequently, cells were washed and transferred to YES medium with the same concentrations of Dip and L-ornithine for an additional 3 h. Pull down assays were performed as previously described (Jacques et al., 2014), with the following modifications. The cells were disrupted with glass beads in lysis buffer A containing 50 mM HEPES (pH 7.5), 140 mM NaCl, 5 mM MgCl_2_, 1 mM EDTA, 1% Triton X-100, 0.1% sodium deoxycholate, 1 mM phenylmethylsulfonyl fluoride, 50 mM sodium fluoride, 0.2 mM sodium orthovanadate, and a mixture of protease inhibitors (Sigma-Aldrich, P8340). Cell lysates (~2.0 mg of total proteins per sample) were incubated with IgG-Sepharose 6 Fast-Flow beads (GE Healthcare), and the suspensions were mixed end-over-end on a rotating wheel for 2 h at 4°C. The beads underwent four washes. The first wash used a modified buffer A containing 500 mM NaCl instead of 140 mM. The second and third washes were performed with buffer B containing 10 mM Tris–HCl (pH 8.0), 250 mM lithium chloride, 0.5% NP-40, 0.5% sodium deoxycholate, and 1 mM EDTA. Prior to the final wash, samples were transferred to fresh microtubes, and the last wash was carried out using buffer C containing 10 mM Tris–HCl (pH 8.0) and 1 mM EDTA. Subsequently, the attached complexes were dissociated from the beads using an elution buffer consisting of 50 mM Tris–HCl (pH 8.0), 10 mM EDTA, and 1% SDS. Further dissociation occurred at 65°C in a ThermoMixerC (Eppendorf) with a rotating speed of 1,200 rpm for 12 min. The resulting immunoprecipitated fractions were mixed with an SDS loading buffer containing urea (4.0 M) and thiourea (1.0 M), heated at 95°C for 5 min, and then resolved by electrophoresis on 8% SDS-polyacrylamide gels. Proteins were electroblotted onto nitrocellulose membranes and immunodetection assays were performed as described previously (Brault et al., 2022).

### Detection of Fc

The extraction method for isolating Fc from *S. pombe* cells has been previously described (Plante and Labbé, 2019; Brault et al., 2022). Following their isolation and lyophilization, Fc from the indicated strains was resuspended in water and applied onto preheated silica gel 60 F_254_ thin-layer chromatography (TLC) plastic sheets (EMD Millipore). TLC assays were conducted using a solvent containing 80% aqueous methanol. Commercially purified holo-Fc (Sigma-Aldrich) served as the positive control for signal detection.

### Fluorescence microscopy

Fluorescence and differential interference contrast images (Nomarski) of cells were captured using a Nikon Eclipse E800 epifluorescence microscope (Nikon, Melville, NY) outfitted with a Hamamatsu ORCA-ER digital cooled camera (Hamamatsu, Bridgewater, NJ). The cells were observed at 1,000× magnification using an excitation wavelength of 465–495 nm for detecting GFP signal. The representative cell fields depicted in this study are derived from a minimum of five independent experiments. Furthermore, displayed cell fields represent protein localization in 200 cells examined from GFP-expressing strains.

### Accession numbers

The UniProt knowledgebase (UniProtKB) amino acid sequences for *S. pombe* Sib3, *U. maydis* Fer5, *M. tuberculosis* MbtK/Rv1347c, *A. fumigatus* SidL and SidF are Q9UUE3, Q4PEN1, P9WK15, Q4WJX7, and Q4WF55, respectively.

## Results

### The His^248^ residue of Sib3 is essential for enabling cells to grow under low-iron conditions and to synthesize Fc

**Figure 1:**
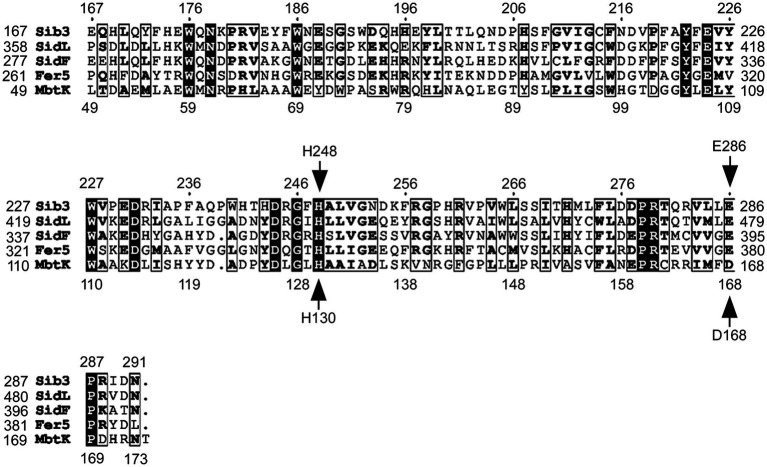
Sequence alignment of Gcn5-related N-acetyltransferase (GNAT) domains of selected Sib3 orthologs. Amino acid alignment of *Schizosaccharomyces pombe* Sib3 predicted acetyltransferase GNAT-like domain with other known GNAT-like domains found in proteins that participate in siderophore biosynthesis, including *Aspergillus fumigatus* SidL and SidF, *Ustilago maydis* Fer5, and *Mycobacterium tuberculosis* Mbtk. Arrows indicate two conserved amino acid residues that were found to be critical for acetyltransferase activity in *M. tuberculosis* Mbtk protein. These His^130^ and Asp^168^ residues in Mbtk corresponds to His^248^ and Glu^286^ residues in *S. pombe* Sib3.

Our previous findings have shown that Sib3 is required for Fc production in *S. pombe* (Brault et al., 2022). Through comparisons of amino acid sequences, Sib3 was predicted to contain a Gcn5-related N-acetyltransferase (GNAT) domain (Rutherford et al., 2024). The presence of this domain suggested that Sib3 has the capability to catalyze the transfer of an acetyl group to N^5^-hydroxyornithine, resulting in the production of N^5^-acetyl-N^5^-hydroxyornithine, which is involved in Fc biosynthesis. Alignment of an amino acid region (residues 167–291), encompassing the putative GNAT-like domain of Sib3, with four other predicted or known siderophore-biosynthetic acetyltransferases, revealed a high degree of conservation for His^248^ and Glu^286^ (Figure 1). In the case of Glu^286^, its conservation was greater with the three other fungal acetyltransferases from *A. fumigatus* (SidL and SidF) and *U. maydis* (Fer5) compared to that of the bacterium *M. tuberculosis* Rv1347c (MbtK). In the case of His^248^, it corresponds to residue His^130^ in the *M. tuberculosis* Rv1347c protein (Figure 1) (Frankel and Blanchard, 2008). In *M. tuberculosis*, mutagenesis of His^130^ blocks the acetyltransferase reaction by Rv1347c, disrupting its activity and consequently siderophore biosynthesis. As for *S. pombe* Glu^286^, the corresponding residue is Asp^168^ in *M. tuberculosis*. According to the predicted positioning of amino acid side chains within the Rv1347c active site, it has been suggested that the Asp^168^ residue of Rv1347c may enhance the basicity of His^130^ or stabilize the proximal protonated imidazolium ion to optimize proper coordination of the substrate for its acetylation (Frankel and Blanchard, 2008).

**Figure 2:**
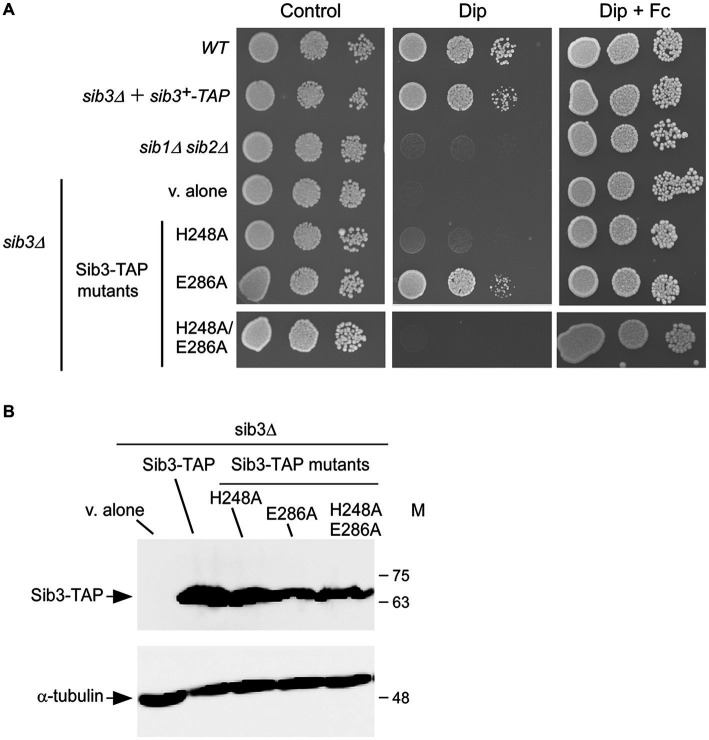
The His^248^ residue is required for Sib3 function. **(A)** Wild-type (*WT*), *sib1Δ*, *sib2Δ*, and *sib3Δ* cells containing an empty vector (*v. alone*), and *sib3Δ* cells expressing either *sib3^+^-TAP*, *sib3H248A-TAP*, *sib3H286A-TAP*, or *sib3H248A/E286A-TAP* allele were spotted in serial dilutions (4,000 cells/10 μL; 400 cells/10 μL; and 40 cells/10 μL) onto YES medium. This medium was prepared without Dip or Fc supplementation (control) or supplemented with Dip (130 μM) or a combination of Dip and Fc (10 μM). All plates were incubated for 4 days at 30°C, and photographed. **(B)** *sib3Δ* cells containing an empty vector (*v. alone*), or expressing the indicated alleles were grown to an OD_600_ of 1.0, and subsequently treated with Dip (130 μM) for 90 min. Whole cell extract preparations were analyzed using immunoblot assays with anti-mouse IgG and anti-α-tubulin antibodies. The positions of the molecular weight standards (in kDa) are indicated on the right side.

Our previous findings have shown that *S. pombe* cells lacking Sib3 (*sib3Δ*) are unable to grow on YES medium in the presence of the iron chelator Dip (Brault et al., 2022). Given the requirement of *M. tuberculosis* His^130^ and Asp^168^ residues for the catalytic activity of the bacterial acetyltransferase, we initiated experiments to examine whether mutations in the His^248^ and Glu^286^ residues of Sib3 affected the *S. pombe*’s ability to grow under iron-limiting conditions. Spot assays were performed using *sib3Δ* cells expressing either TAP-tagged *sib3^+^* or TAP-tagged mutant versions of *sib3*, where either His^248^, Glu^286^ or both His^248^ and Glu^286^ had been substituted by Ala residues. As shown in Figure 2A, *sib3Δ* cells expressing the *sib3H248A-TAP* or *sib3H248A/E286A-TAP* allele exhibited a severe growth defect when spotted on iron-depleted medium compared to the isogenic wild-type strain or *sib3Δ* cells expressing *sib3^+^-TAP*. In contrast, *sib3Δ* cells expressing the *sib3E286A-TAP* allele exhibited a robust growth equivalent to the *sib3Δ* mutant strain expressing *sib3^+^-TAP* (Figure 2A). As negative controls, a *sib1Δ sib2Δ* strain or *sib3Δ* cells containing an empty integrative vector (v. alone) failed to grow on YES medium supplemented with Dip (Figure 2A).

Consistent with the involvement of Sib3 in Fc biosynthesis, the results showed that growth of *sib3Δ* cells expressing TAP-tagged Sib3H248A and Sib3H248A/E286A mutants or an empty vector was rescued when exogenous Fc (10 μM) was added in YES medium (Figure 2A). To ensure that all TAP epitope-tagged *sib3* alleles were expressed in *sib3Δ* cells, steady-state protein levels of Sib3 and its mutant derivatives were analyzed by immunoblotting. The results showed that all these proteins were expressed in the *sib3Δ* strain (Figure 2B). Taken together, these results indicate that Sib3 His^248^ is required for *S. pombe* growth in iron-poor media without Fc supplementation.

### The Sib3 His^248^ is required for cross-feeding between Fc-producing *Schizosaccharomyces pombe* cells and *Saccharomyces cerevisiae* fet3Δ arn1-4Δ cells expressing Arn1-GFP

**Figure 3:**
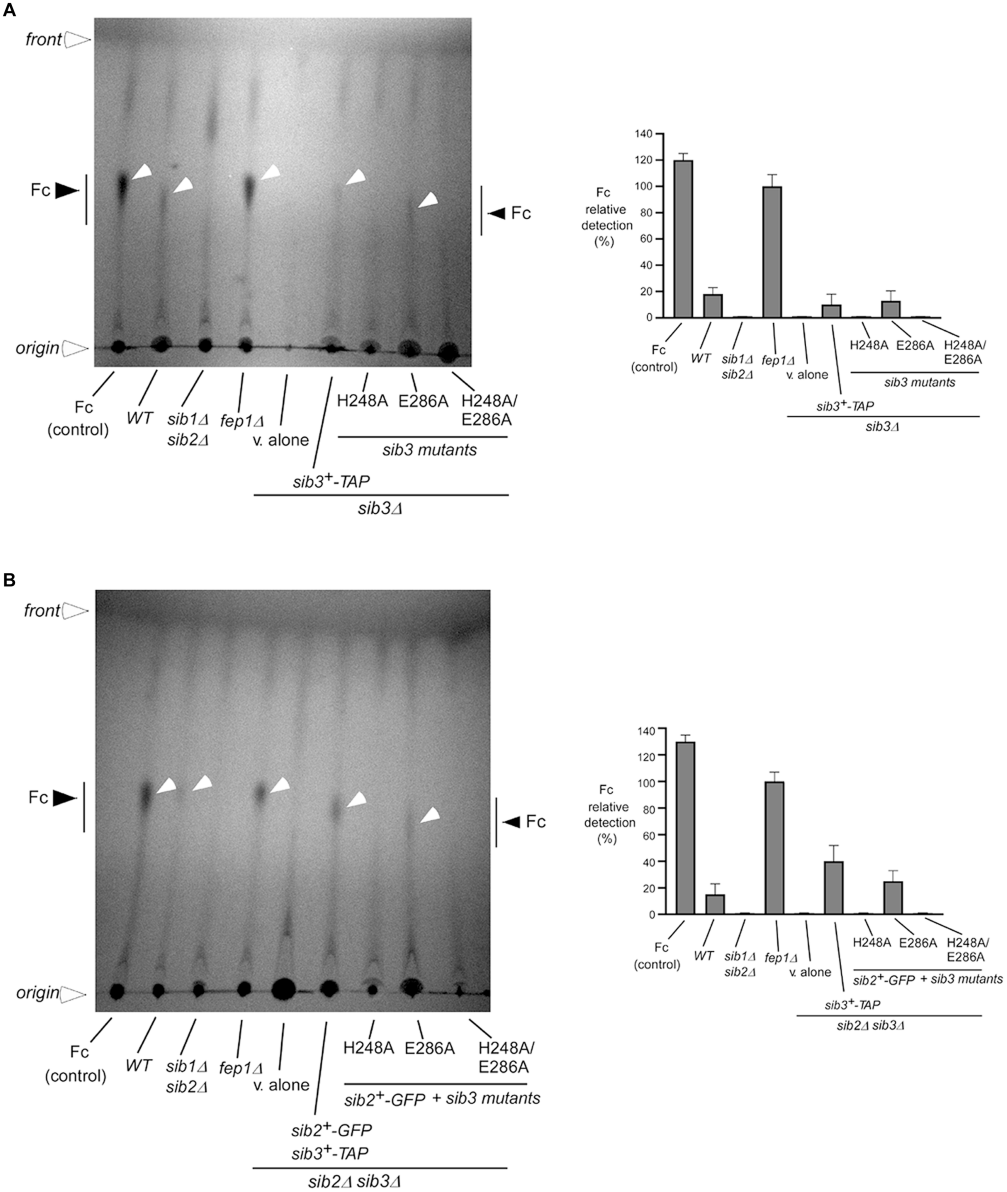
The Sib3 His^248^ is needed for Fc biosynthesis. **(A)** Wild-type (*WT*), *sib1Δ sib2Δ*, *fep1Δ*, and *sib3Δ* strains were used to assess Fc production. In the case of *sib3Δ* cells, they either carried an empty plasmid (*v. alone*) or expressed one of the following alleles: *sib3^+^-TAP*, *sib3H248A-TAP*, *sib3E286A-TAP*, or *sib3H248A/E286A-TAP*. All strains were grown to an OD_600_ of 0.5 in YES medium and then treated with Dip (100 μM) for 5 h. Total Fc was extracted and analyzed by TLC on silica gel sheets. Commercially purified Fc (15 μg) (*control*) was loaded as a reference. Black and white arrowheads indicate the migration of Fc, whereas open arrowheads show the origin of sample loading and front of gel migration. **(B)** A similar set of strains was cultured under the same growth conditions as in *panel* **(A)**, except that the *sib2Δ sib3Δ* double mutant strain was used instead of the *sib3Δ* single mutant strain. When specified, *sib2^+^-GFP*-expressing *sib2Δ sib3Δ* cells containing the indicated wild-type *sib3^+^-TAP* or *sib3-TAP* mutant alleles were used for whole-cell extract preparations and detection of Fc by TLC. Graphical representations of the quantification for five independent TLC assays are shown (right side of each panel). Values are the averages of five determinations ± standard deviations.

Given the findings that His^248^ of Sib3 was required to promote cell growth under low-iron conditions, we sought to determine whether Fc was produced in *sib3Δ* mutant cells expressing the *sib3H248A-TAP* or *sib3H248A/E286A-TAP* allele. Whole cell extracts were prepared from the wild-type, *sib1Δ sib2Δ*, *fep1Δ*, *sib3Δ*, and *sib2Δ sib3Δ* strains. Similarly, extracts were isolated from *sib3Δ* cells expressing *sib3^+^-TAP* and its mutant derivatives or *sib2Δ sib3Δ* cells co-expressing *sib2^+^-GFP* and *sib3^+^-TAP* or *sib3-TAP* mutated alleles. When Fc was isolated from extracts of the wild-type strain, a weak but reproducible Fc signal was detected by thin-layer chromatography (TLC) (Figures 3A,B). The detection of the Fc signal was stronger in extracts isolated from a *fep1Δ* strain due to the absence of Fep1 and, therefore, the lack of Fep1-mediated transcriptional repression of *sib1^+^* and *sib2^+^* genes (Figures 3A,B) (Mercier and Labbé, 2010; Brault et al., 2022). In contrast, extract preparations from *sib3Δ*, *sib1Δ sib2Δ*, and *sib2Δ sib3Δ* mutant strains exhibited no detectable Fc signal (Figures 3A,B). In the case of *sib3Δ* and *sib2Δ sib3Δ* strains expressing *sib3H248A-TAP* and *sib3H248A/E286A-TAP* mutant alleles, their extract preparations were devoid of detectable Fc (Figures 3A,B). Conversely, Fc signals were detected in extract preparations from *sib3Δ* cells expressing *sib3^+^-TAP* and *sib3E286A-TAP* or from *sib2Δ sib3Δ* cells co-expressing *sib2^+^-GFP* and *sib3^+^-TAP* or *sib3E286A-TAP* alleles (Figures 3A,B). Together, these results indicated that the His^248^ residue of Sib3 is essential for its activity in Fc production.

Our previous studies have shown that wild-type *S. pombe* cells can support the growth of *S. cerevisiae* cells by secreting Fc-iron as the sole source of iron (Brault et al., 2022). This Fc-dependent cross-feeding between *S. pombe* and *S. cerevisiae* requires *S. pombe* cells competent in Fc biosynthesis, thus expressing functional Sib1, Sib2, and Sib3 proteins. For *S. cerevisiae*, its ability to acquire exogenous Fc is revealed by co-culturing a *S. cerevisiae fet3Δ arn1-4Δ* mutant, in which the Fc-iron transporter *ARN1* gene is reintroduced to mediate the uptake of Fc (Brault et al., 2022).

**Figure 4:**
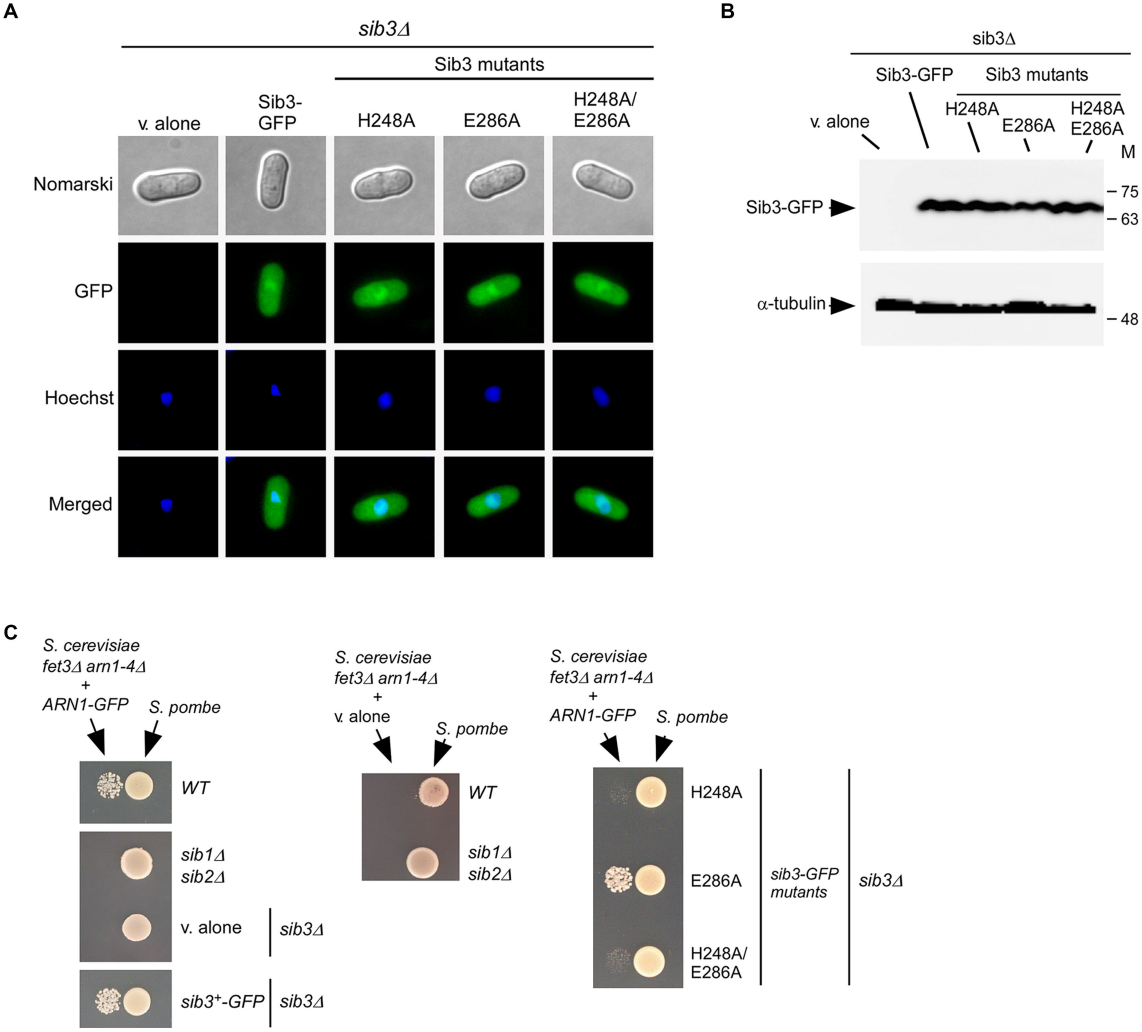
The His^248^ residue of *Sib3* is required to promote Fc-dependent growth of *Saccharomyces cerevisiae fet3Δ arn1-4Δ* cells expressing ARN1. **(A)** *sib3Δ* cells expressing GFP-tagged Sib3 and its mutant derivatives, or harboring an empty integrative plasmid (*v. alone*) were examined by fluorescence microscopy for the presence of GFP-dependent fluorescence signals (*center top*). Cell morphology was monitored using Normarski optics (*far top*). The cells were treated with Hoechst for nuclear DNA staining (*center bottom*). The merged images are shown in the *far bottom* panels. The results from microscopy represent findings from five independent experiments. **(B)** Whole cell extracts from aliquots of cultures examined in *panel* **(A)** were analyzed by immunoblotting using anti-GFP and anti-α-tubulin antibodies. Positions of the molecular weight standards (in kDa) are shown to the right. **(C)** The indicated *Schizosaccharomyces pombe* strains were cultured to an OD_600_ of 1.0 in the presence of FeCl_3_ (10 μM). At this stage, half of cells (1 × 10^7^ cells/10 μL) were spotted (right side of each pair of spots) onto SD^-Cu-Fe^ medium. *Saccharomyces cerevisiae fet3Δ arn1-4Δ* cells containing an empty vector (*v. alone*) or expressing *ARN1-GFP* were grown in SD^-Cu-Fe^ medium to an OD_600_ of 1.0. Subsequently, the cells were washed, diluted 10,000-fold, and spotted (3,000 cells/10 μL) (left side of each pair of spots) onto SD^-Cu-Fe^ medium in the vicinity of the *S. pombe* strains.

Studies have shown that a functional Sib3-GFP protein is constitutively expressed and distributed throughout the cytoplasm and nucleus of cells (Matsuyama et al., 2006; Brault et al., 2022). To verify that mutant derivatives of Sib3 exhibited the same cellular localization as the wild-type protein, microscopic analyses of GFP-tagged Sib3H248A, Sib3E286A, and Sib3H248A/E286A proteins were performed alongside Sib3-GFP. Results showed that Sib3-GFP and its mutant derivatives exhibited similar fluorescence signal patterns, being present in both the cytoplasm and nucleus (Figure 4A). Whole-cell extracts from aliquots of the cultures used for microscopic analyses were subjected to immunoblot assays. The results showed that Sib3-GFP and its mutant variants were expressed, as detected through immunoblot analysis (Figure 4B).

We next assessed the impact of Sib3 mutants on the ability of *S. pombe* to promote the growth of ARN1-expressing *S. cerevisiae fet3Δ arn1-4Δ* cells in co-culture experiments. An *S. pombe sib3Δ* strain expressing Sib3-GFP, Sib3H248A-GFP, Sib3E286A-GFP, or Sib3H248A/E286A-GFP was grown to logarithmic phase under iron-replete conditions (10 μM FeCl_3_). At this point, cell cultures were spotted (1 × 10^7^ cells/10 μL) onto a copper-and iron-poor medium, designated SD^-Cu-Fe^. *Saccharomyces cerevisiae fet3Δ arn1-4Δ* cells expressing a functional Arn1-GFP protein were cultured in SD^-Cu-Fe^ medium until logarithmic phase and then diluted 10,000-fold before being point-inoculated in the vicinity of the indicated S. pombe strains. The results showed that growth of the *S. cerevisiae fet3Δ arn1-4Δ ARN1-GFP* strain was severely limited when co-cultured with an *S. pombe sib3Δ* strain expressing *sib3H248A-GFP* or *sib3H248A/E286A-GFP* allele, due to the lack of Fc production (from *S. pombe*) necessary to fuel the growth of *S. cerevisiae* cells (Figure 4C). In contrast, growth of *S. cerevisiae fet3Δ arn1-4Δ ARN1-GFP* cells was supported when point-inoculated near the wild-type *S. pombe* strain or *S. pombe sib3Δ* cells expressing *sib3^+^-GFP* or *sib3E286A-GFP* allele (Figure 4C). As negative controls, *S. pombe sib1Δ sib2Δ* and *sib3Δ* cells containing an empty vector were unable to promote the growth of *S. cerevisiae fet3Δ arn1-4Δ* cells expressing Arn1-GFP (Figure 4C). Similarly, an *S. cerevisiae fet3Δ arn1-4Δ* mutant strain lacking Arn1-GFP was unable to grow in the vicinity of the wild-type *S. pombe* and *S. pombe sib1Δ sib2Δ* strains (Figure 4C). Collectively, these results indicated that the mutation of Sib3 His^248^ leads to cross-feeding inhibition between *S. pombe* and *S. cerevisiae fet3Δ arn1-4Δ* cells expressing Arn1-GFP.

### The interaction between Sib2 and Sib3 persists even when His^248^ and Glu^286^ residues of Sib3 are changed to alanines

**Figure 5:**
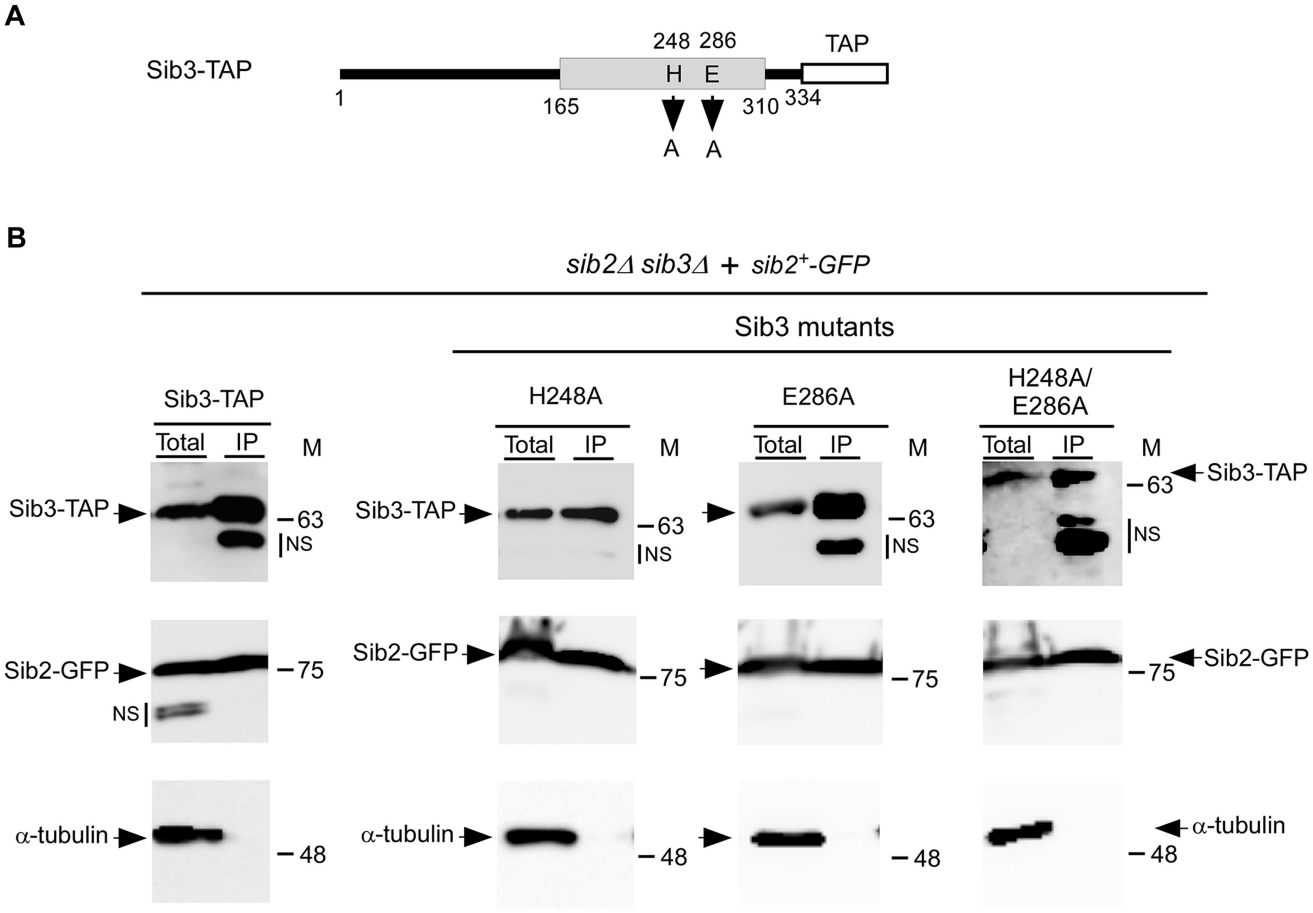
Wild-type Sib3-TAP and its mutant derivatives harboring substitutions at residues His^248^, Glu^286^, or His^248^ and Glu^286^ are found in a complex with Sib2. **(A)** Schematic representation of Sib3-TAP with amino acid substitutions at residues 248 and 286. The gray box indicates the predicted location of a GNAT-like domain, whereas the white box represents the TAP tag. The amino acid sequence numbers refer to the position relative to the first amino acid of Sib3. **(B)** *sib2Δ sib3Δ* cells co-expressing GFP-tagged Sib2 and TAP-tagged Sib3 or one of the mutant derivatives, either Sib3H248A, Sib3E286A, or Sib3H248A/E286A. The indicated cultures were grown to the mid-logarithmic phase and incubated in the presence of Dip for 90 min. Whole cell extracts (Total) were then incubated with IgG-Sepharose beads. The immunoprecipitated fractions (IP) were analyzed by immunoblot assays using anti-mouse IgG, anti-GFP, and anti-α-tubulin antibodies. Aliquots of total protein lysates were probed with the same antibodies to ascertain the presence of epitope-tagged proteins prior to incubation with beads. The positions of the molecular weight standards (in kDa) are indicated on the right side of the panels. NS stands for non-specific.

Our previous studies have shown that functional Sib2-GFP and Sib3-TAP fusion proteins interact to form a protein complex (Brault et al., 2022). Considering the necessity of the His^248^ residue for Sib3 function in Fc biosynthesis, we investigated whether this amino acid is required for the association between Sib2 and Sib3. Furthermore, we examined the impact of substituting the Glu^286^ residue with an alanine on the Sib2-Sib3 association in parallel experiments (Figure 5A). We first created a *sib2Δ sib3Δ* mutant strain, wherein the *sib3^+^-TAP*, *sib3H248A-TAP*, *sib3E286A-TAP*, or *sib3H248A/E286A-TAP* allele was co-expressed with the *sib2^+^-GFP* allele. Cells co-expressing each combination of protein pairs were cultured to the mid-logarithmic phase and then incubated in the presence of Dip for 90 min. Subsequently, whole-cell extracts were prepared, and immunoprecipitation was performed using IgG-Sepharose beads, which specifically bound to TAP-tagged Sib3 and its mutant derivatives. Immunoblot analysis of proteins retained by the beads indicated the presence of both Sib3-TAP and Sib2-GFP in the immunoprecipitated fraction (IP) (Figure 5B). In the case of the IP fractions from cells co-expressing Sib3H248A-TAP and Sib2-GFP, Sib3E286A-TAP and Sib2-GFP, or Sib3H248A/E286A-TAP and Sib2-GFP, the results showed that Sib2-GFP remained an interacting partner of these Sib3 mutant derivatives (Figure 5B). The specificity of coimmunoprecipitation assays was validated by the absence of α-tubulin in the IP fractions (Figure 5B). Together, these results revealed that substituting the His^248^ and Glu^286^ residues with alanines in Sib3 does not hinder the ability of Sib3 to associate with Sib2.

### Sib3 interacts with two distinct regions of Sib2

**Figure 6:**
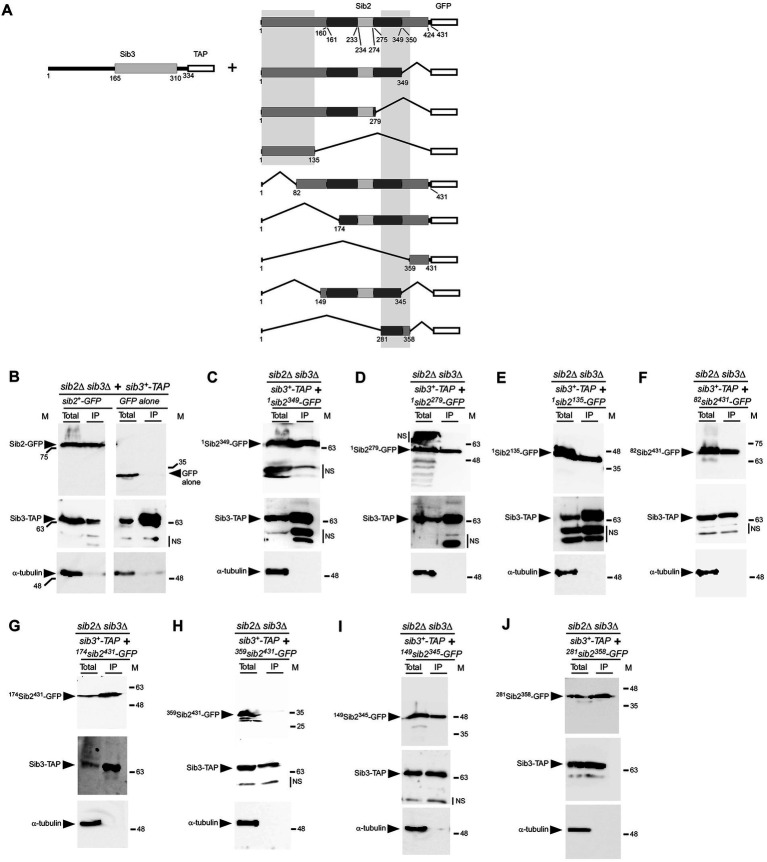
Regions of *Sib2* involved in association with *Sib3*. **(A)** Diagram illustrating truncated versions of Sib2. Two regions predicted to bind FAD encompass residues 1–160 and 350–424 of Sib2, whereas Sib2 161–233 and Sib2 275–349 regions are predicted to be involved in NADPH binding. Ornithine binding to Sib2 is predicted to involve residues 234–274 and Lys^64^ and Ser^402^. The white box indicates GFP fused to Sib2. The two gray vertical boxes indicate two distinct minimal regions of Sib2 required for interaction with Sib3. For Sib3, the gray box indicates the predicted location of GNAT-like domain, whereas the white box represents the TAP tag. Amino acid sequence numbers refer to positions relative to the first amino acid of the Sib2 and Sib3 proteins, respectively. **(B)** As positive and negative controls, *sib2Δ sib3Δ* cells co-expressing *sib2^+^-GFP* and *sib3^+^-TAP*, or *GFP* alone and *sib3^+^-TAP*, were grown to an OD_600_ of 1.0 and then treated with Dip (250 μM) for 90 min. Whole cell extracts (Total) were subjected to immunoprecipitation (IP) using IgG-Sepharose beads. The bound fractions were eluted and analyzed by immunoblot assays using anti-GFP, anti-mouse IgG, and anti-α-tubulin antibodies. **(C–J)** *sib2Δ sib3Δ* cells co-expressing *sib3^+^-TAP* and the indicated truncated forms of *sib2^+^-GFP* were grown under the same conditions used in *panel* **(B)**. After preparation of cell extracts (Total), samples were subjected to IP as performed in *panel* **(B)**. Unbound and bound fractions were analyzed with the same antibodies as mentioned above. The positions of the molecular weight standards (in kDa) are indicated on the left and right sides of the panels. NS stands for non-specific.

To gain insight into the regions of Sib2 that interact with Sib3, we created a series of *sib2* alleles encoding truncated versions of Sib2, either from its C-terminal or N-terminal end (Figure 6A). C-terminal deletions of Sib2-GFP were generated by removing 82, 152, and 296 amino acid residues, resulting in the creation of ^1^Sib2^349^-GFP, ^1^Sib2^279^-GFP, and ^1^Sib2^135^-GFP mutants, respectively. Each of these mutants was co-expressed with full-length Sib3-TAP in *sib2Δ sib3Δ* cells, which were then treated with Dip for 3 h. Moreover, *sib2Δ sib3Δ* cells were used to co-express *sib3^+^-TAP* and *GFP* alleles under the same growth conditions. Cell lysates were extracted from all these cultures and then incubated with IgG-Sepharose beads. Results showed that ^1^Sib2^349^-GFP, ^1^Sib2^279^-GFP, and ^1^Sib2^135^-GFP truncated proteins co-immunoprecipitated with TAP-tagged Sib3, whereas GFP alone was consistently absent in the IP fraction (Figures 6B–E). For N-terminal deletions of Sib2-GFP, they were generated by deleting 81, 173, and 358 amino acid residues, resulting in the production of ^82^Sib2^431^-GFP, ^174^Sib2^431^-GFP, and ^359^Sib2^431^-GFP mutants, respectively. Furthermore, two additional truncated versions of Sib2 were created by combining N-and C-terminal deletions of the protein. These mutant versions of Sib2 were designated as ^149^Sib2^345^-GFP, and ^281^Sib2^358^-GFP, respectively (Figure 6A). *sib2Δ sib3Δ* cells co-expressing the aforementioned mutated forms of Sib2-GFP with full-length Sib3-TAP were incubated in the presence of Dip (250 μM). After 3 h, whole-cell extracts were prepared and subjected to co-immunoprecipitation assays using IgG-Sepharose beads. Results showed that the IP fractions contained ^82^Sib2^431^-GFP, ^174^Sib2^431^-GFP, ^149^Sib2^345^-GFP, and ^281^Sib2^358^-GFP proteins, which were identified as interacting partners with Sib3-TAP (Figures 6F–J). In contrast, co-immunoprecipitation assays failed to reveal an interaction between ^359^Sib2^431^-GFP and Sib3-TAP (Figure 6H). Notably, α-tubulin was not detected in the IP fractions (Figures 6B–J), indicating the specificity of the pulldown assays. Taken together, these results highlight two minimal regions of Sib2 implicated in its association with Sib3: one encompassing residues 1–135 and the other spanning residues 281–358.

### The C-terminal segment of Sib3 from amino acids 290–334 is required for its interaction with Sib2

**Figure 7:**
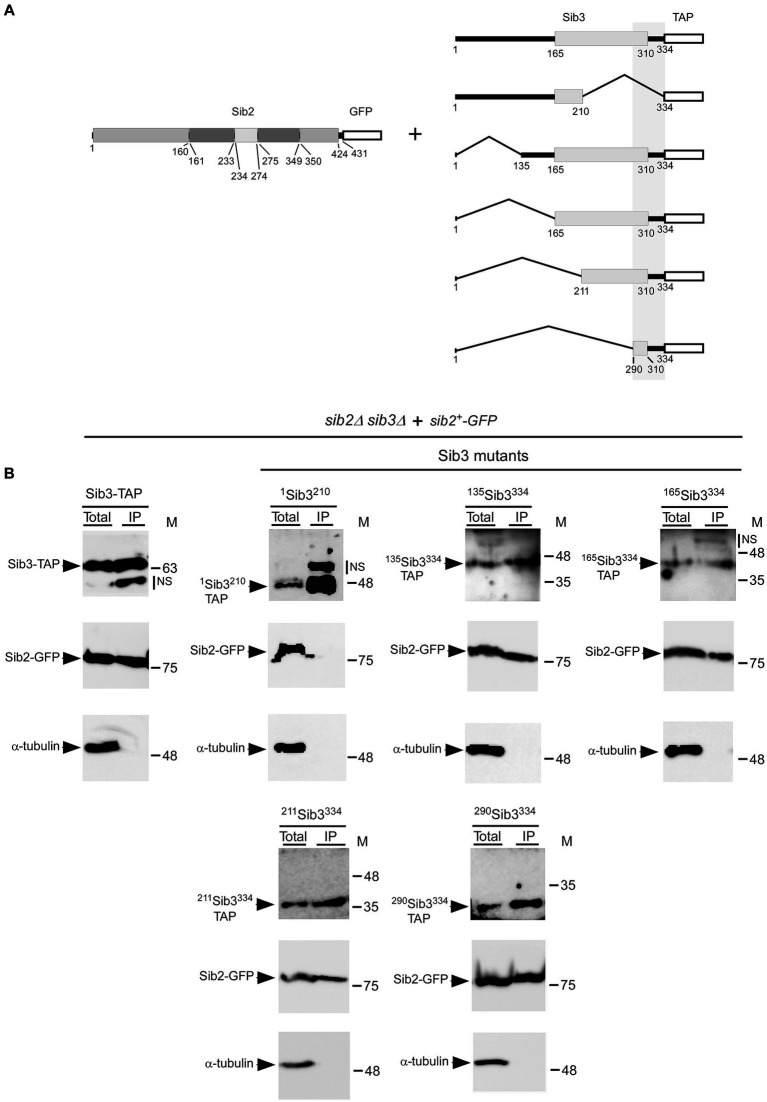
Minimal C-terminal region of Sib3 required for interaction with Sib2. **(A)** Schematic representation of truncated versions of Sib3. The predicted GNAT-like domain corresponds to residues 165–310 of Sib3, whereas the white box represents the TAP tag. The vertical gray box highlights a minimal region of Sib3 for interaction with Sib2. Full-length Sib2 is depicted with its putative FAD- (residues 1–160 and 350–424), NADPH- (residues 161–233 and 275–349), and ornithine-binding (residues 234–274) domains. Amino acid sequence numbers refer to positions relative to the first amino acid of the Sib2 and Sib3 proteins, respectively. **(B)** *sib2Δ sib3Δ* cells harboring GFP-tagged Sib2 and TAP-tagged Sib3 or its mutant derivatives were cultured to an OD_600_ of 1.0. Cultures were then incubated in a medium containing Dip (250 μM) for 90 min. Cell lysates (Total) were incubated with IgG-Sepharose beads. After washings, the bound fractions were analyzed by immunoblotting using anti-GFP, anti-mouse IgG, and anti-α-tubulin antibodies. The positions of the molecular weight standards (in kDa) are indicated on the right side of the panels. NS stands for non-specific.

Our experiments on Sib2 prompted us to determine the region on Sib3 required for interacting with Sib2. Truncations were created from both the C-and N-terminal ends of Sib3-TAP (Figure 7A). The first construct removed the last 124 amino acids of Sib3 (^1^Sib3^210^-TAP). Subsequent constructs removed the first 134, 164, 210, and 289 amino acids of Sib3, designated as ^135^Sib3^334^-TAP, ^165^Sib3^334^-TAP, ^211^Sib3^334^-TAP, and ^290^Sib3^334^-TAP, respectively. Following this, TAP pull-down experiments were performed using protein lysates from cells co-expressing full-length Sib2-GFP with either wild-type Sib3-TAP, ^1^Sib3^210^-TAP, ^135^Sib3^334^-TAP, ^165^Sib3^334^-TAP, ^211^Sib3^334^-TAP, or ^290^Sib3^334^-TAP. Results consistently showed the presence of Sib2-GFP as an interacting partner with wild-type Sib3-TAP in the IP fraction (Figure 7B). In contrast, the interaction between ^1^Sib3^210^-TAP and Sib2-GFP ceased when the C-terminal region of Sib3, encompassing residues 211 to 334 was deleted, as Sib2-GFP was absent in the IP fraction. On the other hand, Sib2-GFP was detected in the IP fraction containing the other truncated forms of Sib3-TAP from the N-terminal end, including ^135^Sib3^334^-TAP, ^165^Sib3^334^-TAP, ^211^Sib3^334^-TAP, and ^290^Sib3^334^-TAP. Taken together, these results indicated that the minimal C-terminal region of Sib3 encompassing amino acids 290–334 is sufficient for Sib3-Sib2 association.

## Discussion

Fc biosynthesis in *S. pombe* relies on Sib1, Sib2, and Sib3. Although the first two proteins were identified some time ago (Schrettl et al., 2004a), Sib3 has only been discovered recently (Brault et al., 2022). Based on its primary amino acid sequence, Sib3 is a putative member of the GNAT protein family, which includes 38 proteins in the *S. pombe* database (Rutherford et al., 2024). Among them, Sib3 is the only one that contains a MbtK/IucB-like domain, typically found in siderophore-biosynthetic transacetylase enzymes (Haas et al., 2008). As previously reported, we demonstrated that disruption of the *sib3^+^* gene (*sib3Δ*) prevents Fc biosynthesis (Brault et al., 2022). This is consistent with the predicted second enzymatic step of Fc biosynthesis, in which the transacetylase Sib3 is required for the acetylation of N^5^-hydroxyornithine to N^5^-acetyl-N^5^-hydroxyornithine. This modified compound then combines with three glycine residues to form the finished Fc product through the action of Sib1.

Comparison of the amino acid sequence of Sib3 with those of two other fungal acetyltransferases, SidL (*A. fumigatus*) and Fer5 (*U. maydis*), as well as the bacterial acetyltransferase Rv1347c (*M. tuberculosis*), revealed that the polypeptide sequence encompassing the GNAT domain was the most highly conserved region among these proteins. In particular, the His^248^ of Sib3 exhibits a high degree of conservation with His^130^ in the Rv1347c protein, which is known to be essential for its acetyltransferase activity (Frankel and Blanchard, 2008). Consistently, when the His^248^ of Sib3 was mutated to Ala, *S. pombe* cells expressing this *sib3H248A* allele were unable to produce Fc and then failed to grow under iron-starved conditions. Moreover, an *S. pombe sib3H248A* mutant ceased to provide Fc to *S. cerevisiae fet3Δ arn1-4Δ ARN1* cells when the two yeast species were spotted in close proximity, leading to cross-feeding inhibition between *S. pombe* and *S. cerevisiae fet3Δ arn1-4Δ ARN1* cells.

In the case of Rv1347c, its Asp^168^ residue is proposed to play a critical role in coordinating substrate entry prior its acetylation (Frankel and Blanchard, 2008). Asp^168^ of Rv1347c corresponds to Glu^286^ in *S. pombe* Sib3. However, our results showed that *S. pombe sib3Δ* or *sib2Δ sib3Δ* cells expressing the *sib3E286A* allele in the presence of *sib2^+^* or *sib2^+^-GFP* produced Fc at a comparable level to that detected in wild-type or *sib3Δ* cells expressing *sib3^+^-TAP* or *sib2Δ sib3Δ* cells co-expressing *sib2^+^-GFP* and *sib3^+^-TAP*. Unlike Asp^168^ of Rv1347c, the Glu^286^ to Ala mutation did not interfere with Fc production in the case of *S. pombe* Sib3. This result suggests that Glu^286^ of Sib3 is dispensable for the coordination of N^5^-hydroxyornithine prior its acetylation.

In *A. fumigatus*, disruption of the *sidL* gene (*sidLΔ*) encoding the N^5^-hydroxyornithine acetyl-CoA-N^5^-transacetylase orthologous to Sib3, prevents ferricrocin biosynthesis under iron-replete conditions (Blatzer et al., 2011). However, a *sidLΔ* mutant remains competent to produce ferricrocin under iron-limiting conditions, highlighting the existence of a second, uncharacterized N^5^-hydroxyornithine acetyl-CoA-N^5^-transacetylase that is expressed to replace for the loss of SidL (*sidLΔ*) under low-iron conditions. In *S. pombe*, inactivation of sib3^+^ (*sib3Δ*) completely inhibited Fc biosynthesis, irrespective of cellular iron status. We therefore conclude that, in contrast to *A. fumigatus*, *S. pombe* lacks a second N^5^-hydroxyornithine acetyl-CoA-N^5^-transacetylase, as a *sib3Δ* null strain failed to produce Fc under both iron-replete and iron-limiting conditions.

After replacing His^248^, Glu^286^ or both residues in Sib3 with alanines, we tested whether these substitutions affected the interaction between Sib3 and Sib2. The results showed that the His^248^ residue, which is supposed to act as a catalytic base in the predicted acyltransfer reaction by Sib3, is not involved in the ability of Sib3 to interact with Sib2. This is because the mutant proteins Sib3H248A and Sib3H248A/E286A still form a Sib2-Sib3 heteroprotein complex, as detected by coimmunoprecipitation assays. A similar result was observed in cells expressing the *sib3E286A* allele. Therefore, we conclude that the His^248^ and Glu^286^ residues are not required for the association between Sib3 and Sib2.

In our previous study, we found that the expression of *sib3^+^* is constitutive and remains constant regardless of the presence or absence of the iron-responsive GATA-type transcriptional repressor Fep1 (Brault et al., 2022). In contrast, the situation is different for the *sib1^+^* and *sib2^+^* genes, their mRNA levels are induced in response to iron starvation and repressed under iron-replete conditions in a Fep1-dependent manner (Mercier and Labbé, 2010; Brault et al., 2022). Although siderophore biosynthetic genes are generally transcriptionally regulated in response to changes in iron levels, there are few exceptions, including the *npgA* and *sidL* genes in *A. nidulans*, which are constitutively expressed like *sib3^+^* in *S. pombe* (Oberegger et al., 2003; Blatzer et al., 2011; Brault et al., 2022). A common point between Sib3 and SidL is that both are N^5^-hydroxyornithine acetyl-CoA-N^5^-transacetylases. Their constitutive expression may ensure their continued presence to avoid any breaking point in the Fc-and ferricrocin-biosynthetic pathways, respectively. Alternatively, Sib3 and SidL may catalyze additional acetyltransfer reactions that are not solely devoted to siderophore biosynthesis. This latter possibility may explain why Sib3 is observed throughout the cells and not exclusively in the cytoplasmic compartment like Sib1 and Sib2 (Brault et al., 2022).

Deletion mapping assays of the Sib3-TAP fusion protein have shown that the C-terminal amino acids from residues 290 to 334 are required for the interaction of Sib3 with Sib2. Interestingly, this region was also identified using a predicted three-dimensional model of the Sib3-Sib2 heteroprotein complex generated by AlphaFold-Multimer (Evans et al., 2022). Using this structure prediction system, amino acid residues such as Ser^304^, Leu^307^, Ile^321^, Thr^322^, and Met^325^ within the C-terminal region encompassing amino acids 290–334 of Sib3 have been suggested to play a role in Sib3-Sib2 association. However, the contribution of these residues in the C-terminal region of Sib3 to the interaction between Sib3 and Sib2 must await a comprehensive dissection of this minimal region.

Database analysis revealed that *S. pombe* Sib2 is orthologous to the ornithine N^5^-monooxygenase SidA in *A. fumigatus* (Eisendle et al., 2003; Schrettl et al., 2004b). There is a 35% identity and 58% similarity between Sib2 and SidA. Crystal structures of SidA have been solved, and different domains of the protein have been identified in the context of its tridimensional structure (Franceschini et al., 2012; Campbell et al., 2020). Furthermore, two additional related ornithine hydroxylases, PvdA from *Pseudomonas aeruginosa* and Ktz1 from *Kutzneria* sp. *744*, have been crystallized to elucidate their structures (Olucha and Lamb, 2011; Setser et al., 2014). All three enzymes, SidA, PvdA, and Ktz1 share a conserved fold and adopt the same homotetrameric structure. The protein fold consists of three distinct domains. The FAD-and NADPH-binding domains exhibit characteristics of α/β Rossman-like nucleotide folds, whereas the ornithine-binding domain is small and helical in nature (Franceschini et al., 2012; Campbell et al., 2020). Comparing the predicted model structure of Sib2 to the available crystal structures of SidA, PvdA, and Ktz1 led us to determine that amino acids 113–134 and 307–358 of Sib2 are predicted to be fully accessible and exposed on the outer surface of each Sib2 monomer, which is anticipated to assemble into a tetrameric structure. Deletion mapping analyses revealed that the N-terminal 135 amino acids of Sib2 and the region of Sib2 from residues 281 and 358 are sufficient for interaction with Sib3. Indeed, these two regions contain amino acids 113–134 and 307–358 of Sib2, respectively. Based on these results, it is envisioned that a functional tetramer of Sib2 associates with four Sib3 molecules in a multimeric complex. After Sib2 catalyzes the hydroxylation of ornithine to N^5^-hydroxyornithine, the presence of Sib3 bound to Sib2 would facilitate the next sequential reaction, which consists of the acetylation of N^5^-hydroxyornithine. This Sib2-Sib3 association would optimize the first two steps in the biosynthesis of hydroxamate-containing siderophores.

## Data Availability

The raw data supporting the conclusions of this article will be made available by the authors, without undue reservation.
